# Functional coupling of human pancreatic islets and liver spheroids on-a-chip: Towards a novel human *ex vivo* type 2 diabetes model

**DOI:** 10.1038/s41598-017-14815-w

**Published:** 2017-11-03

**Authors:** Sophie Bauer, Charlotte Wennberg Huldt, Kajsa P. Kanebratt, Isabell Durieux, Daniela Gunne, Shalini Andersson, Lorna Ewart, William G. Haynes, Ilka Maschmeyer, Annika Winter, Carina Ämmälä, Uwe Marx, Tommy B. Andersson

**Affiliations:** 1 TissUse GmbH, Berlin, Germany; 2 Bioscience Diabetes, Cardiovascular and Metabolic Diseases, IMED Biotech Unit, AstraZeneca, Gothenburg, Sweden; 3 DMPK, Cardiovascular and Metabolic Diseases, IMED Biotech Unit, AstraZeneca, Gothenburg, Sweden; 4 Drug Safety and Metabolism, IMED Biotech Unit, AstraZeneca, Cambridge, UK; 5 Department of Physiology and Pharmacology, Section of Pharmacogenetics, Karolinska Institutet, Stockholm, Sweden

## Abstract

Human *in vitro* physiological models studying disease and drug treatment effects are urgently needed as more relevant tools to identify new drug targets and therapies. We have developed a human microfluidic two-organ-chip model to study pancreatic islet–liver cross-talk based on insulin and glucose regulation. We have established a robust co-culture of human pancreatic islet microtissues and liver spheroids maintaining functional responses up to 15 days in an insulin-free medium. Functional coupling, demonstrated by insulin released from the islet microtissues in response to a glucose load applied in glucose tolerance tests on different days, promoted glucose uptake by the liver spheroids. Co-cultures maintained postprandial glucose concentrations in the circulation whereas glucose levels remained elevated in both single cultures. Thus, insulin secreted into the circulation stimulated glucose uptake by the liver spheroids, while the latter, in the absence of insulin, did not consume glucose as efficiently. As the glucose concentration fell, insulin secretion subsided, demonstrating a functional feedback loop between the liver and the insulin-secreting islet microtissues. Finally, inter-laboratory validation verified robustness and reproducibility. Further development of this model using tools inducing impaired glucose regulation should provide a unique *in vitro* system emulating human type 2 diabetes mellitus.

## Introduction

Human long-term organoid cultures in microfluidic microphysiological systems (MPS; or organs-on-a-chip) can emulate human biology and, therefore, enable detailed temporal studies of the physiological functions of organs, organ cross-talk and pharmacological effects of drugs^1,2^. One major goal of MPS efforts is to recapitulate a disease state and study the effects of drug treatment. Such models have the potential to transform drug discovery, enabling efficient studies, and potential identification, of new drug targets, as well as testing of drug interventions in physiologically relevant models. Several recent publications have shown considerable progress in developing relevant human models, such as human single-organ lung-on-a-chip^3^ or liver-on-a-chip platforms^4^. Further development into disease models is progressing for lung equivalents^5^ and for liver equivalents^6–8^.

However, systemic human diseases progress through the disruption of the homeostatic cross-talk of two or more organs. To emulate such systemic interactions, a few microphysiological platforms are aiming to develop on-chip co-cultures of different organoids in separate culture compartments, interconnected through microfluidic channels^9–14^. A first organotypic homeostatic long-term co-culture of human skin biopsies with three-dimensional (3D) human liver spheroids has been established successfully on a multi-organ chip (MOC) platform^15^. Subsequently, MOC-based long-term co-cultures of liver spheroids with human 3D intestinal^16^ and neuronal^17^ tissue models were established for systemic repeated dose substance testing.

Type 2 diabetes mellitus (T2DM) is increasing in incidence and associated with multi-organ co-morbidities^18–20^. As insulin resistance develops, the pancreatic islets meet the increasing demand for insulin by increasing secretion and expanding islet mass. It is only when islets fail to adapt that blood glucose levels increases and overt T2DM develops. As insulin is a key regulator of hepatic metabolism, shifting the balance from glucose production to favour glucose storage, hepatic insulin resistance contributes to impaired glucose homeostasis defining T2DM. A wide range of animal models displaying various characteristics of T2DM has been generated in the past^21^. Despite this, extrapolations from animal research have resulted in a poor translation of studies to understand and improve glucose metabolism, from molecular mechanistic findings through phenotypic manifestation and progression of disease to pharmacological effects of novel therapies^22^. Importantly, many of the anti-diabetic drugs currently in clinical use, though effectively treating symptoms, have limited effects on disease progression. Microphysiological co-culture systems of human pancreas and liver, which are two of the key organs involved in maintaining glucose homeostasis, might provide a unique platform for emulating T2DM. Consequently, our aim was to establish a human two-organ-chip (2-OC) model replicating long-term physiological cross-talk between pancreatic islet microtissues and liver spheroids.

The pancreatic islets of Langerhans are endocrine organelles scattered throughout the exocrine portion of the pancreas. They are comprised of at least five distinct cell types: α cells (secreting glucagon), β cells (insulin), γ/PP cells (pancreatic polypeptide), δ cells (somatostatin) and ε cells (ghrelin). Isolated pancreatic islets represent a useful model for studying islet function and hormone secretion. As of today, MPS have only been used for short-term cultures of pancreatic islet microtissues to evaluate hormone secretion profiles^23,24^ and interaction with endothelial cells^25^. The 3D InSight™ pancreatic islet microtissues (InSphero AG, Switzerland) used in this study are produced from reconstituted dispersed human pancreatic islet cells retaining the composition of α, β and δ cells representative of normal human pancreatic islets. The 3D islet microtissues maintain normal insulin secretion response to a glucose load in long-term culture for up to four weeks^26–28^.

In the liver, insulin stimulates glycogen synthesis and inhibits glycogen breakdown. It also stimulates glycolysis and inhibits gluconeogenesis. In addition to its effects on glucose metabolism, insulin has a variety of anabolic actions in the liver, stimulating lipid synthesis and release, protein synthesis, and inhibiting the breakdown of these substances^29,30^. Several studies have explored various platforms to build human liver organ models^31^. Major efforts in the area have been directed towards drug metabolism and toxicity studies using primary human liver cells or hepatoma cell lines. In this study, we used spheroids formed from HepaRG cells, a carcinoma cell line exhibiting many features similar to primary human hepatocytes, and has been used successfully in drug metabolism and toxicity studies^32,33^. HepaRG cells were also found to be useful in studies inducing steatosis by lipids^34^ or drugs^35^, indicating their usefulness as a metabolic disease model. HepaRG cells have been demonstrated to form 3D spheroids in static culture^36^, with improved functional performance over a culture period of up to 21 days^37^. Furthermore, including extracellular matrix producing stellate cells is essential for the formation and long-term preservation of HepaRG liver spheroids^11^.

We co-cultured human liver spheroids composed of HepaRG cells and primary human stellate cells with human pancreatic islet microtissues to model their physiological cross-talk. In particular we wanted to establish physiological regulation of circulating glucose driven by the biological source of insulin – the pancreatic islets. Reciprocally, we intended to investigate the effect of glucose on insulin secretion. Experiments were conducted in two independent laboratories to establish inter-laboratory reproducibility. The latter is the basis for further development into a reliable human T2DM model.

## Results

### Two-organ-chip and co-culture process design

**Figure 1:**
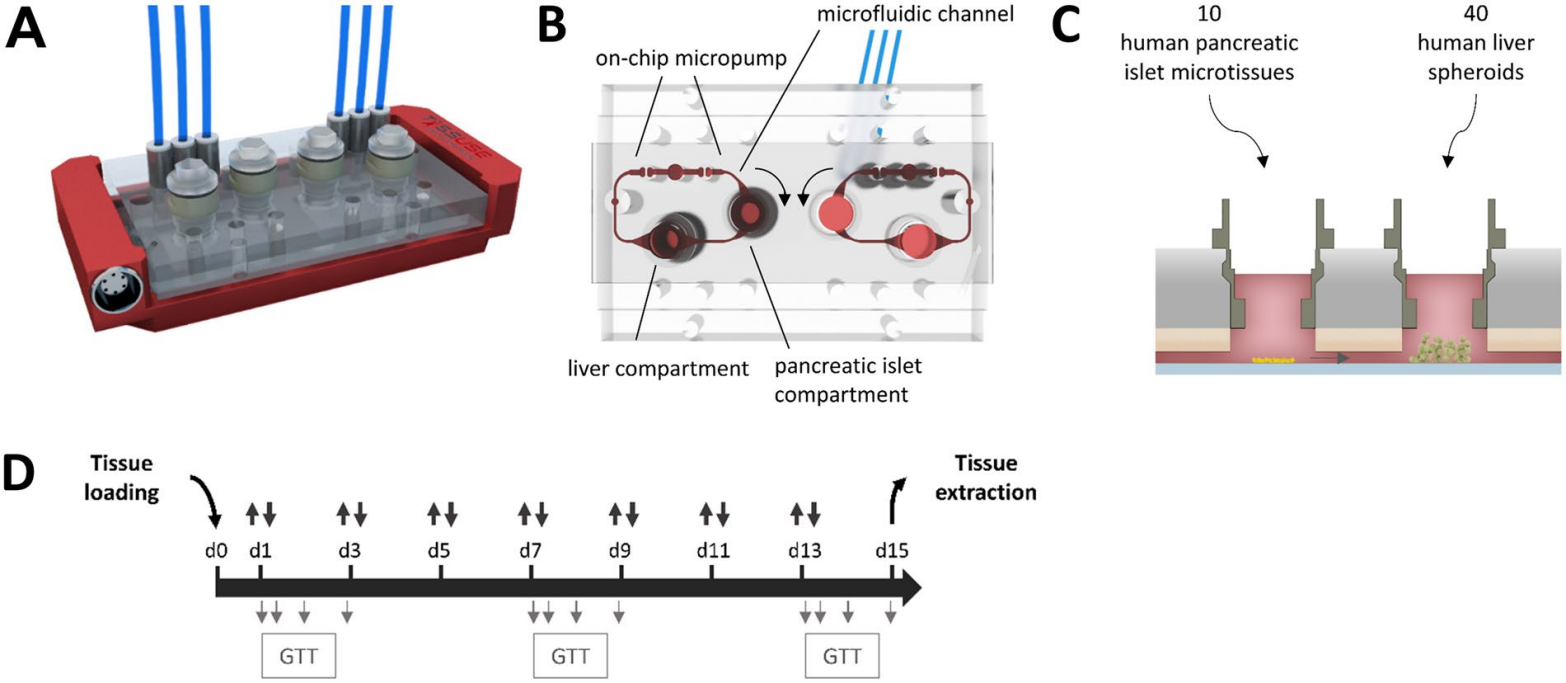
The microphysiological two-organ-chip (2-OC). (**A**) A 3D view of the assembled device including temperature support (red). (**B**) Illustration of the view from underneath with media circuits, respective culture compartments and micropump valves highlighted in red. (**C**) Standard tissue loading scheme of organ equivalents for 2-OC co-culture. (**D**) 15-day co-culture schematic: repeated total media exchange (double arrows, 300 µl from each compartment), additional sampling at 0, 8, 24 and 48 h for a glucose tolerance test (grey arrows, 15 µl from each compartment).

A 2-OC enabling the culture of human pancreatic islet microtissues and liver spheroids was used (Fig. 1A and B). The tissue compartments and the interconnecting microfluidic channel contained a total media volume of 610 µl. A peristaltic on-chip micropump enabled a continuous pulsatile flow with a pumping frequency of 0.475 Hz and a pressure of 500 mbar resulting in an average flow of 4.94 µl/min with a complete media turnover time of approximately 2 h^38^, supporting high tissue perfusion rates at pulsatile flow. Pancreatic islet microtissues and liver spheroids were adapted to the medium routinely used for the liver spheroids devoid of the customary supplement of insulin. Ten pancreatic islet microtissues were placed in the compartment upstream of a chamber containing 40 liver spheroids (Fig. 1C). This is equivalent to a factor of 100,000 present in a normal human pancreas (estimated to contain on the order of one million pancreatic islets). The number of liver spheroids represents a similar fraction of a normal human liver.

A robust 15-day co-culture process was developed and used in two independent laboratories. This included tissue loading, seven complete media exchanges and terminal tissue extraction for further analysis to evaluate the long-term reproducibility of the organ cross-talk (Fig. 1D). Glucose utilisation was evaluated as a measure of organ cross-talk by raising the glucose level in the culture medium to 11 mM mimicking the standard glucose tolerance test (GTT) performed in humans. Subsequently, samples were collected at consecutive time points (Fig. 1D) for measurements of glucose and insulin in the circulating media.

### Characterisation of human pancreatic islet microtissues and human liver spheroids before chip culture

**Figure 2:**
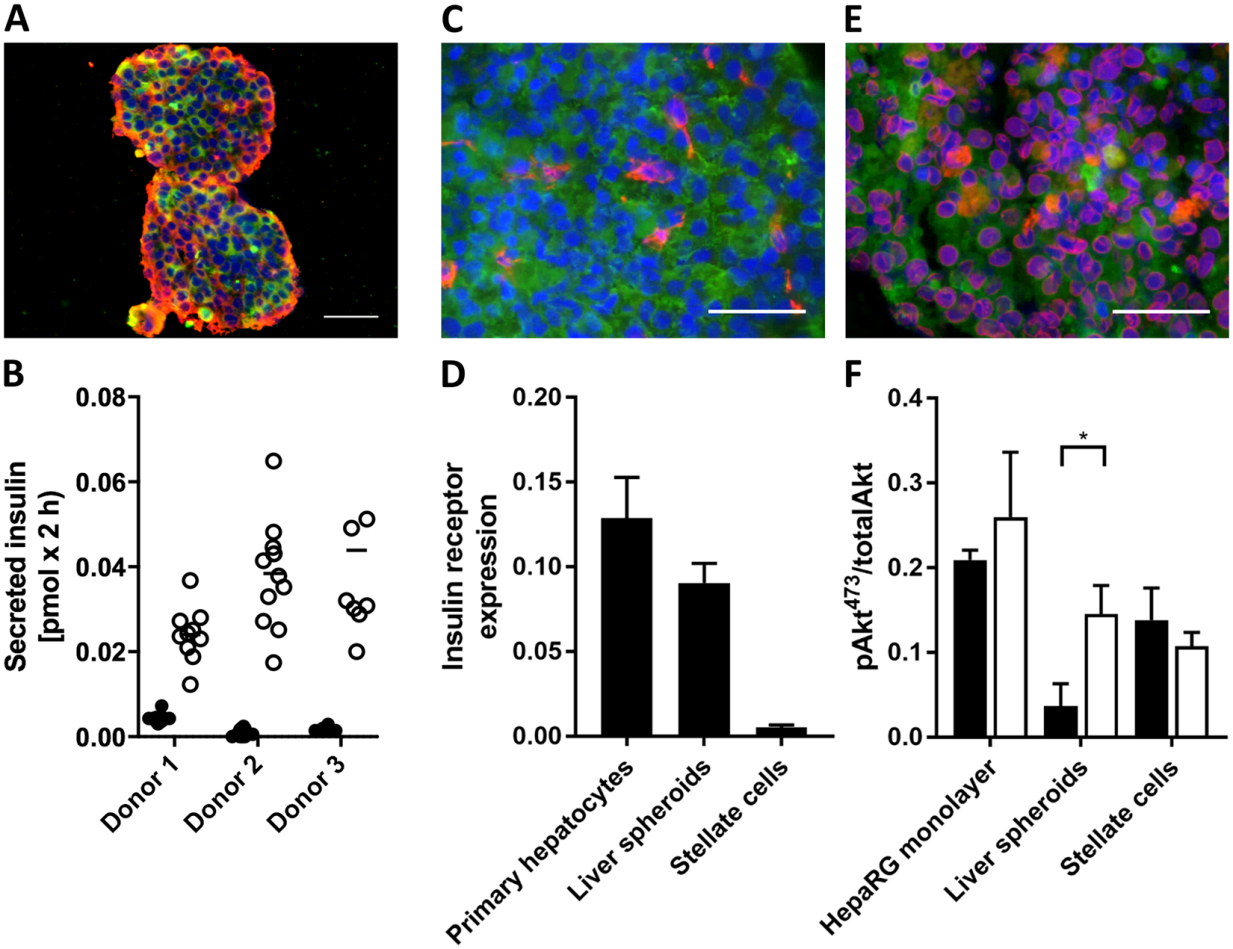
Status of pancreatic islet microtissues and liver spheroids prior to co-culture. (**A**) Protein expression of insulin (red) and glucagon (green) in pancreatic islet microtissues. Nuclei stained with DAPI (blue). Scale bar: 50 µm. (**B**) Glucose-stimulated insulin secretion (GSIS) from single islet microtissues in low (2.8 mM, black dots) and high (16.8 mM, white dots) glucose. (**C**) Protein expression of cytokeratin 8/18 (green) and vimentin (red) in liver spheroids. Nuclei stained with DAPI (blue). Scale bar: 50 µm. (**D**) Insulin receptor mRNA expression in primary human hepatocytes, liver spheroids and stellate cells alone, normalized to TATA-box-binding protein, n = 4 for liver spheroids and stellate cells, n = 2 for primary human hepatocytes. Data are means ± SD. (**E**) Protein expression of albumin (green) and CYP3A4 (red) in liver spheroids. Nuclei stained with DAPI (blue). Scale bar: 50 µm. (**F**) Phosphorylated Akt (pAkt, serine-473) normalized to total Akt in unstimulated (black bars) and insulin-stimulated (1 nM, white bars) conditions. *p < 0.05 using unpaired two-tailed Student t test. Data shown as mean + SD, n = 3.

Both the functionality and morphology of representative organoid equivalents were evaluated before their transfer to the 2-OCs. The presence of insulin-producing β cells and glucagon-producing α cells in the islet microtissues was confirmed by immunohistochemistry (Fig. 2A). In addition, all preparations of islet microtissues (n = 3 donors) were confirmed to secrete insulin in response to glucose (Fig. 2B) prior to use. In liver spheroids, the even distribution of stellate cells expressing vimentin across the hepatocytes expressing cytokeratin 8–18 was shown (Fig. 2C). Furthermore expression of characteristic hepatocyte markers was confirmed by staining for albumin and CYP3A4 (Fig. 2E). The insulin receptor was found to be expressed in liver spheroids at approximately 70% compared to primary human hepatocytes (Fig. 2D). HepaRG cells were the only source for that expression as no expression was detected in stellate cells. Finally, functionality of the liver spheroids was evaluated by measuring Akt phosphorylation as a marker of insulin-receptor signalling (Fig. 2F). No significant difference in Akt phosphorylation was observed between insulin-stimulated (white bars) and unstimulated (black bars) cells in monolayer cultures of either HepaRG cells or stellate cells. By contrast, the spheroid cultures showed an insulin-dependent Akt phosphorylation. Interestingly, this was due to markedly reduced basal Akt activity compared to monolayer cultures (0.04 ± 0.02 vs. 0.21 ± 0.01 pAkt473/totalAkt).

### Long-term functionality of pancreatic islet microtissues in single and co-culture

**Figure 3:**
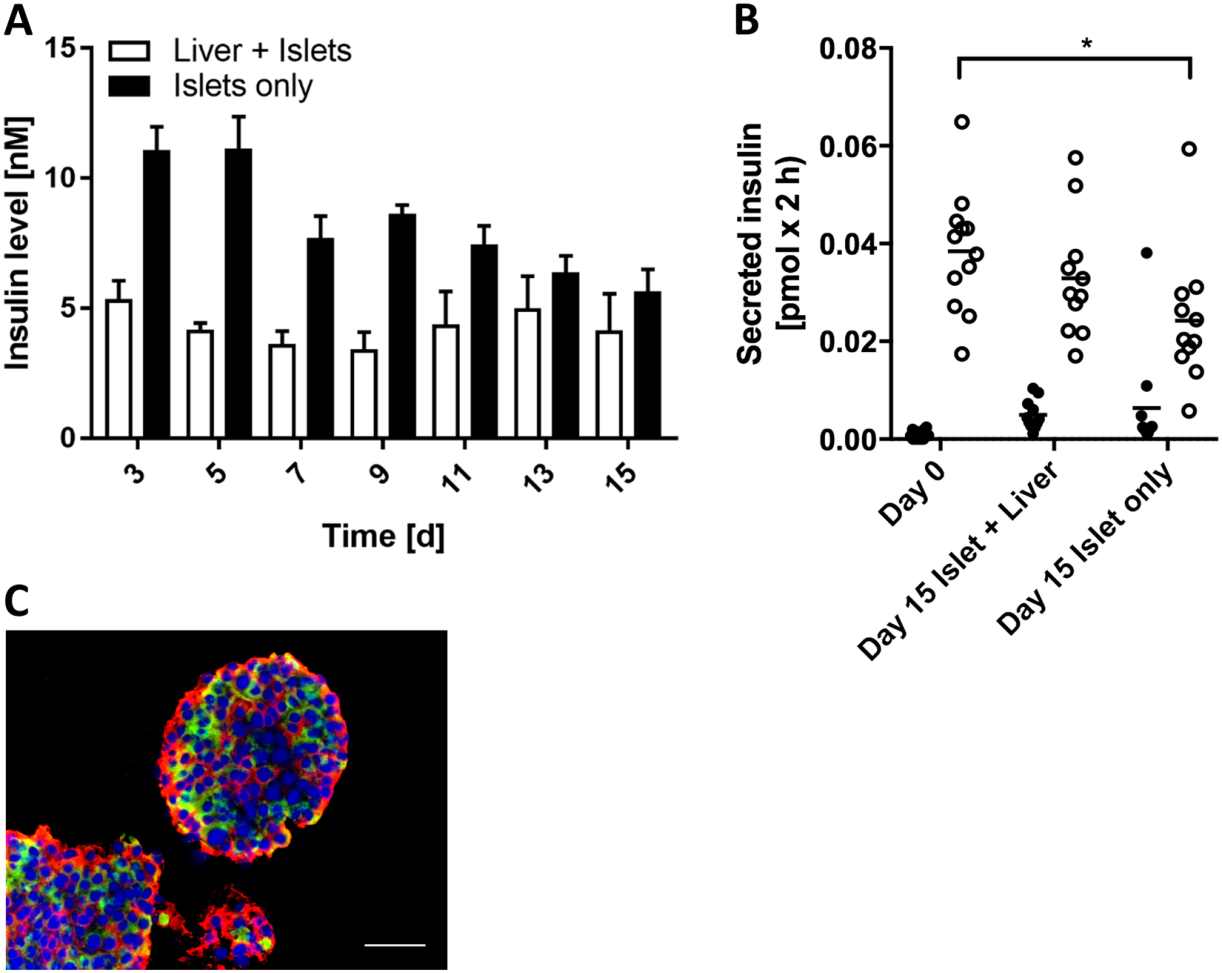
Pancreatic islet microtissues stay functional in co-culture with liver spheroids over 15 days. (**A**) Insulin accumulated over 48-h periods measured in the media of co-cultures and islets alone. Data shown as mean + SD, n = 5. (**B**) GSIS of 11 single islet microtissues in low (2.8 mM, black dots) and high (16.8 mM, white dots) glucose. *p < 0.05 using ANOVA with Tukey’s multiple comparison post hoc test. (**C**) Protein expression of insulin (red) and glucagon (green) in islet microtissues at day 15 in co-culture. Nuclei stained with DAPI (blue). Scale bar: 50 µm.

Following the initial characterisation of the composition and functionality of both islet microtissues and liver spheroids, co-cultures (islets + liver) and single cultures (islets only and liver only) were set up and maintained in the 2-OC for 15 days in a common medium. The co-cultures (Fig. 3A) showed stable, reproducible circulating insulin levels (4.3 ± 1.1 nM) over the entire 15-day culture period. Insulin levels in the islet single cultures were less stable, declining over time with an overall reduction from day 3 (11.08 ± 0.81 nM) to 15 (5.66 ± 0.74 nM) of 49%. Following the 15-day cultivation period, the islet microtissues were removed from the 2-OC and GSIS was measured (Fig. 3B). Whereas the islets maintained in co-culture with liver spheroids maintained glucose stimulated insulin secretion over time, islet microtissues in single cultures had a significantly decreased stimulated insulin secretion compared to day 0. Finally, the islet morphology was not affected in 2-OC culture and both β and α cells were still present with no apparent difference in relative distribution compared to day 0 (Fig. 2C).

### Long-term functionality of liver spheroids in single and co-culture

**Figure 4:**
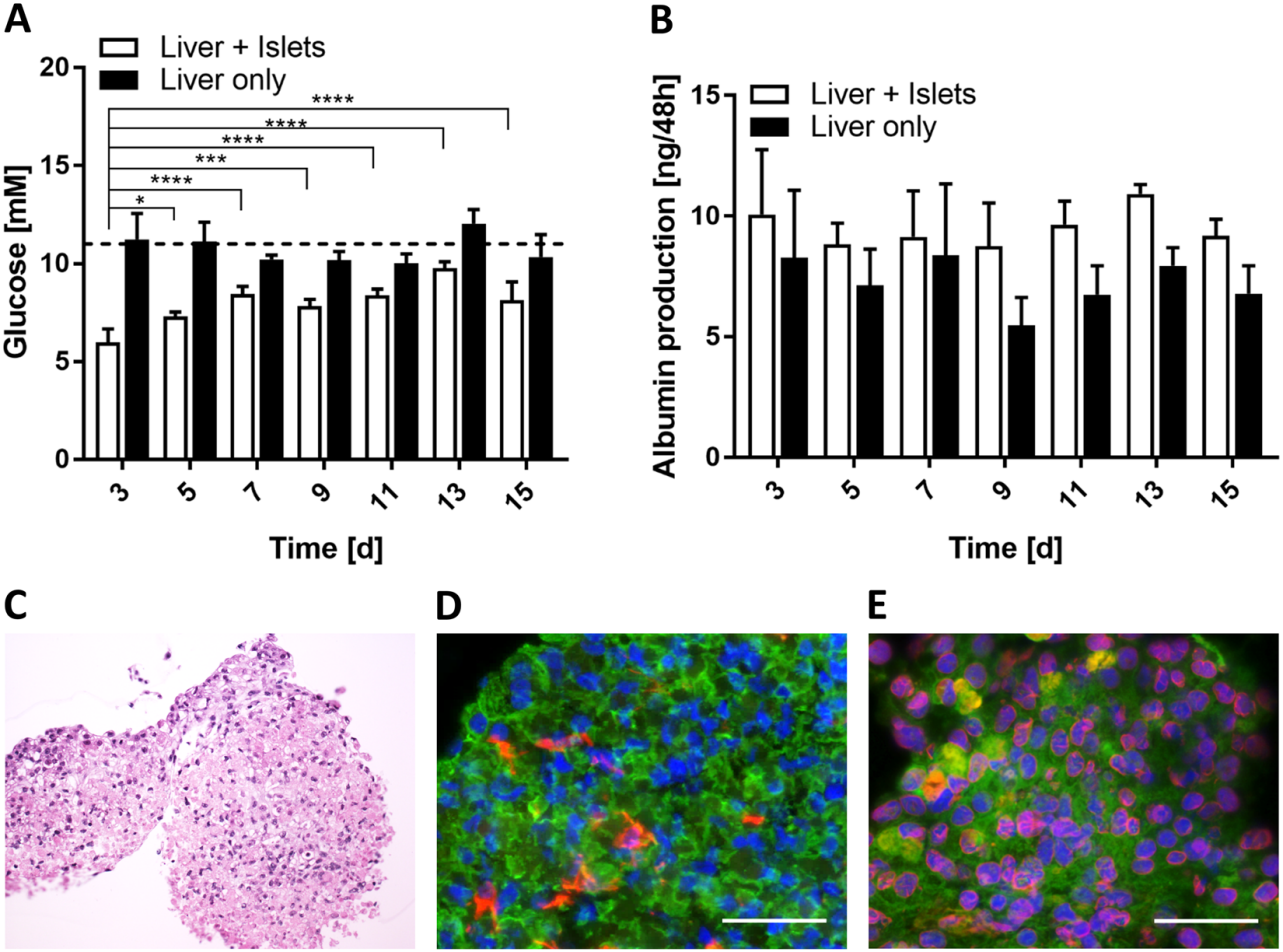
Long-term functionality of liver spheroids. (**A**) Glucose levels 48 h after medium exchange in co-cultures compared to liver spheroids alone. Dotted line indicates glucose concentration of the medium applied. *p < 0.05, ***p < 0.001, ****p < 0.0001 using ANOVA with Dunnett’s multiple comparison post hoc test. Data shown as mean + SD, n = 5. (**B**) Albumin production over 48 h in co-cultures (white bars) and single cultures (black bars). Data shown as mean + SD, n = 5. (**C**) Liver spheroids stained with haematoxylin and eosin after 15 days of co-culture. (**D**) Protein expression of cytokeratin 8/18 (green) and vimentin (red) in liver spheroids after 15 days of co-culture. (**E**) Protein expression of albumin (green) and CYP3A4 (red) in liver spheroids after 15 days of co-culture. Nuclei stained with DAPI (blue). Scale bar: 50 µm.

Insulin secretion by the islet microtissues promoted glucose utilisation by the liver spheroids, demonstrating functional cross-talk between the tissues in co-culture. In single culture, the liver spheroids maintained a stable circulating glucose concentration close to 11 mM (the concentration in the culture medium) over the 15 days in the 2-OC (Fig. 4A, black bars). In co-culture with islets (white bars), on the other hand, the circulating glucose level was reduced within 48 h to a concentration equivalent to normal postprandial levels in man (e.g. 5.9 ± 0.6 mM on day 3), indicating that insulin released by the islet microtissues stimulated glucose uptake by the liver spheroids. However, the ability of the liver spheroids to utilise glucose in the co-cultures decreased over time relative to day 3, but remained stable from day 5 to 15 at levels of 8.3 ± 0.87 mM. Albumin in the circulating media was measured to address whether the reduction in glucose utilisation was due to an impaired function of the liver spheroids. Albumin was produced stably (9.5 ± 1.5 ng in 48 h) in co-cultures and did not correlate with the decreased glucose consumption. Moreover, the histology was inconspicuous and showed intact tissue morphology at day 15 (Fig. 4C). Stellate cell and HepaRG ratios were unchanged compared to day 0 (Fig. 4D), as was albumin and CYP3A4 expression (Fig. 4E).

### On-chip cross-talk between pancreatic islet microtissues and liver spheroids

**Figure 5:**
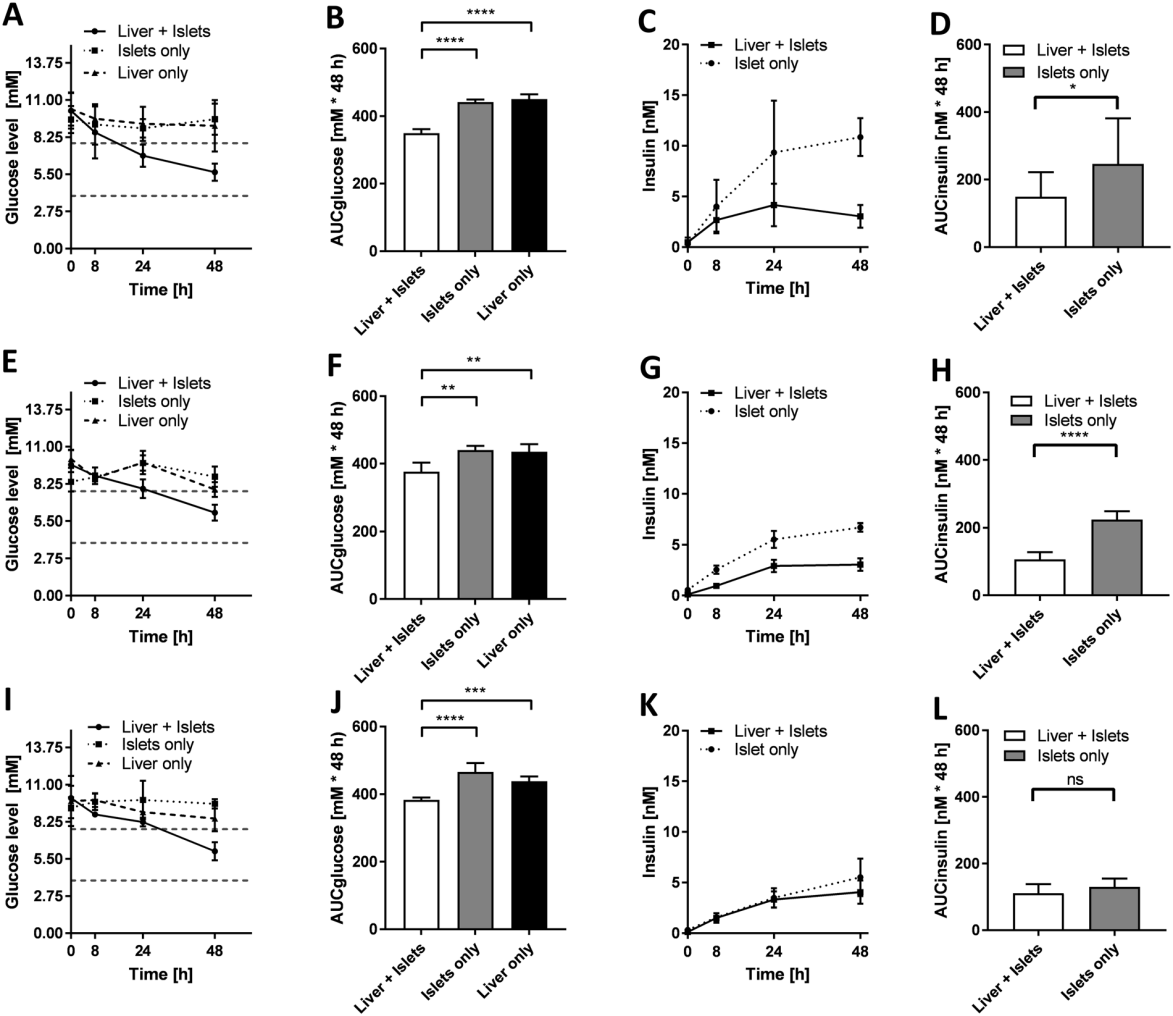
Functional cross-talk between liver spheroids and pancreatic islet microtissues. Glucose level during *in vitro* GTT initiated on day 1 (**A**), 7 (**E**) and 13 (I). Dotted lines indicate physiological postprandial glucose range (3.9–7.8 mM). Area under the glucose response curve for day 1 (**B**), 7 (F) and 13 (J). Insulin level during *in vitro* GTT initiated on day 1 (**C**), 7 (G) and 13 (K). Area under the insulin response curve for day 1 (**D**), 7 (H) and 13 (L). *p < 0.05, **p < 0.01, ***p < 0.001, ****p < 0.0001 using ANOVA with Tukey’s multiple comparison post hoc test. Data shown as mean + SD collected from n = 14 from three independent experiments with three donors of pancreatic islets run at two different laboratories in the first lane and n = 5 from one pancreatic islet donor in the second and third lane.

The response to a high glucose load was studied in more detail on day 1, 7 and 13 of culture to investigate the impact of insulin secretion on glucose utilisation. An *in vitro* GTT was initiated by instantaneously raising the glucose levels to 11 mM by a complete medium exchange. Samples were taken immediately (0 h) and after 8, 24 and 48 h, only causing a reduction of culture volume of less than 10% in total. In contrast to single cultures, the glucose level in co-cultures decreased from the hyperglycaemic to a more normoglycaemic range (3.9 to 7.8 mM) within 24 h on the first day of GTT. Subsequently, glucose levels were balanced in the physiological range without dropping to a hypoglycaemic state. Without the insulin-stimulated glucose uptake, glucose levels in single cultures (islets only and liver only) remained within the hyperglycaemic range (Fig. 5A). Differences in glucose utilisation between co-cultures and single cultures were estimated by calculating the area under the glucose response curve (Fig. 5B), which was significantly decreased in the co-cultures.

Insulin levels in the circulating media during hyperglycaemia rose steadily in the co-cultures and reached a steady state once glucose levels had dropped to normoglycaemic levels (Fig. 5C). Islet microtissue single cultures had a significantly higher insulin secretion (AUC) over 48 h than co-cultures (Fig. 5D). The islet–liver cross-talk observed on day 1 was highly reproducible across experiments with islet microtissues from three different donors performed in two independent laboratories.

Islet–liver cross-talk was maintained over the entire culture period, with similar trends in glucose utilisation and insulin secretion on day 7 and 13. However, compared to day 1, glucose concentrations reached the normoglycaemic range later, between 24 and 48 h after glucose load. Single cultures never reached the normoglycaemic glucose levels at any of the time points tested (Fig. 5A,E and I). The area under the glucose response curves for the co-cultures were significantly reduced compared to single cultures at all time points (Fig. 5F and J). Insulin concentration profiles in the co-cultures were as well comparable to day 1 showing an increase between 0 and 24 h, and a steady state between 24 and 48 h (Fig. 5G and K). The ability of the islet single cultures to maintain insulin secretion in hyperglycaemia decreased with time, leading to no significant difference in the area under the insulin response curve between co-cultures and single cultures on day 13 (Fig. 5L).

## Discussion

T2DM is a multi-organ disease. Disease phenotype and response to treatment effects are dependent on both fully metabolically functional organs and a relevant interplay between them. Here we present a model of organ cross-talk between pancreatic islet microtissues and liver spheroids, two key organs involved in glucose homeostasis. We have used 3D organoids, which are the smallest fully functional building blocks of the corresponding human organs, that are genetically encoded to self-assemble in a proper environment. The islets and the lobules are the functional units of the human pancreas (endocrine portion) and human liver, respectively. The cross-talk between islets and liver is an important mechanism contributing to normal regulation of blood glucose levels. When stimulated by glucose, the pancreatic islets secrete hormones that drain into the hepatic portal system, where glucagon and insulin affects the liver either to take glucose up for fuel storage as glycogen, or to produce and release glucose as a fuel source to support peripheral organs. We here exposed liver spheroids matching ten human liver lobules to ten pancreatic islet microtissues matching one million pancreatic islets in a MPS. Thereby, the respective human counterpart organs – the endocrine part of the pancreas and liver – were downscaled by a factor of 100,000. This allometric scaling will support quantitative *in vitro* to *in vivo* extrapolations using physiologically based modelling and kinetic studies of pharmacology.

The present study resulted in a novel and robust microfluidic chip-based co-culture of human pancreatic islet microtissues and liver spheroids with reproducible organ cross-talk maintained over a period of 15 days in a standardized insulin-free cell culture medium. We established a surrogate functional GTT for the pancreatic islet–liver co-culture simulating the standard intravenous GTT; the gold standard method to simultaneously evaluate insulin secretion and glucose elimination in humans^39–41^. Functional coupling of liver and pancreatic islets was evaluated by measuring the cumulative insulin in media released from the islet microtissues in response to a glucose load. Insulin increased glucose uptake by the liver spheroids accelerating the rate of glucose disappearance from the media. Glucose always returned to normal fasting levels in the co-cultures, but not in the single organ cultures (Fig. 3A,E and I). Thus, insulin secreted into the circulation stimulated glucose uptake by the liver spheroids, while the liver alone did not consume glucose as efficiently. As glucose levels fell, the accumulation of insulin in the media decreased and by 48 h, both glucose and insulin had reached steady state levels, supporting a homeostatic feedback loop between islet microtissues and liver spheroids. Exposed to high glucose over time, islet microtissues cultured in the absence of liver spheroids had reduced ability to release insulin, indicating that the prolonged hyperglycaemia impaired islet function. The results, thus, indicate that the glucose regulation in the 2-OC reflects human glucose homeostasis with adequate insulin response with islet functionality only maintained in co-cultures. However, kinetics of glucose disposal in the 2-OC was considerably slower than *in vivo*, most likely due to the absence of the major glucose-consuming organs and tissues, such as muscle^42^, brain^43^ and kidney^44^, as well as endothelial^45^, stromal and immune^46^ cells in the chip. Furthermore, the media turnover time of 2 h in the 2-OCs might have had an impact on that delay.

Furthermore, we hypothesise that already the hyperglycaemic periods of < 24 h could have induced a lipid-associated insulin resistance in the co-cultured liver spheroids, leading to the decline in glucose utilisation observed over time. In particular, Davidson *et al*.^47^ have demonstrated an onset of insulin resistance in primary human hepatocytes maintained in 25 mM glucose within six days of culture. Furthermore, increased lipid accumulation was seen already at 12.5 mM glucose.

Immunohistological staining before and after co-culture showed that pancreatic islet microtissues and liver spheroids maintained normal morphology with stable expression of insulin and glucagon in the pancreatic islets (Figs 2A and 3C) and Cytokeratin 8/18, vimentin, albumin and CYP3A4 in the liver spheroids (Figs 2C,E and 4D,E) over the entire co-culture period. Furthermore, these images depict a stable distribution of the different cell types of the heterologous organoid equivalents – α and β cells in pancreatic islet microtissues and HepaRG and stellate cells in the liver spheroids – suggesting viable but non-proliferating cells in the spheroids. Even distribution of vimentin, an extracellular matrix protein expressed by stellate cells, illustrates continuous connective tissue activity in liver spheroids during the 15-day co-culture. In addition, the stable secretion of albumin and retained CYP3A4 expression support a physiological phenotype of the liver spheroids. Activation of AKT by insulin via the insulin receptor in the HepaRG spheroids containing stellate cells prior to chip loading demonstrated the physiologically relevant responsiveness of the liver spheroids (Fig. 2F). The ratio between stimulated and unstimulated AKT phosphorylation was considerably higher in the liver spheroids compared to monolayer HepaRG cells, showing that the 3D culture setting is advantageous for a physiological response.

These results now encourage us to induce T2DM conditions in the co-culture, subsequently leading to characteristics of T2DM in the 2-OC model established. The islet–liver culture presented here can, thus, be used to unravel mechanisms and comorbidities associated with T2DM disease progression including β-cell failure, hepatic insulin resistance, steatosis, steatohepatitis and cirrhosis. An additional development of the MOC model would be to include other organs, such as kidney, cardiac tissue and fat, or different combinations of organs, dependent on the scientific issues being investigated. A future improvement of the model would be to replace the HepaRG cells with primary human hepatocytes to reflect the functions of a human liver better and to select specific pheno- and genotypes.

## Methods

### Two-organ-chip design and fabrication

The microphysiological 2-OC, described earlier by Schimek *et al*.^48^, consists of two spatially separated compartments for the cultivation of organ equivalents (Fig. 1B), which are interconnected by a microfluidic channel. A pulsatile flow is established through an on-chip micropump (adapted from Wu *et al*.^49^), enabling cross-talk between the organ equivalents.

Fabrication of the 2-OC was performed as described by Wagner *et al*.^15^. Briefly, replica moulding of polydimethyl-siloxane was applied in a 2-mm high layer containing the respective microfluidic channel, micropump and openings for culture compartments. It is sealed at the bottom by permanent bonding to a glass microscope slide (Menzel, Braunschweig, Germany) using low pressure plasma oxidation (Femto; Diener, Ebhausen, Germany), thus, forming a fluid-tight channel with a height of 100 µm.

The on-chip micropump was actuated by pressured air or vacuum, resulting in a consecutive lowering and raising of the 500-µm thick elastic membranes.

### Cell sources and maintenance

Differentiated HepaRGs (Lot HPR116080 or HPR116189) were obtained from Biopredic International (Rennes, France). Primary human hepatic stellate cells (HHSteC) were purchased from BioreclamationIVT (Westbury, NY, USA). The 3D pancreatic islet microtissues were purchased from InSphero (Schlieren, Switzerland)^27^.

The differentiated HepaRGs were thawed and seeded confluently four days before spheroid formation. Standard HepaRG culture medium consisted of William’s Medium E (PAN-Biotech, Aidenbach, Germany) supplemented with 10% foetal calf serum (Corning, Lowell, MA, USA), 5 µg/ml human insulin (PAN-Biotech), 2 mM L-glutamine (Corning), 5 × 10^−5^ M hydrocortisone hemisuccinate (Sigma–Aldrich, St. Louis, MO, USA), 50 µg/ml Gentamycin Sulfate (Corning) and 0.25 µg/ml Amphotericin B (Corning) or was purchased directly from Biopredict. An amount of 0.5% dimethyl sulfoxide (Carl Roth GmbH, Karlsruhe, Germany) was added to the medium to keep the HepaRGs in a differentiated state. On the following day, the medium was renewed with HepaRG medium containing 2% dimethyl sulfoxide. The cells were maintained in this medium for three days until spheroid formation.

HHSteC (Lot PFP) were expanded in Stellate Cell Medium, provided by ScienCell (Carlsbad, CA, USA). Cells were used between passage 3 and 6. Pre-culture was started at least two days before spheroid formation.

Upon the arrival of pancreatic islet microtissues in 96-well GravityTRAP^TM^ plates, the microtissues were treated according to the manufacturer’s protocol and maintained for two to five days in human islet maintenance medium (InSphero) before transfer to the 2-OC.

### Liver spheroid production

Human liver spheroids were formed combining differentiated HepaRG cells and HHSteC using 384-well spheroid microplates (Corning) in HepaRG medium containing physiological amounts of insulin (1 nM). Briefly, 50 µl containing 24,000 hepatocytes and 1,000 HHSteC was pipetted into each well of the spheroid plate. The plate was centrifuged for 1 min at 300 g and incubated at 37 °C and 5% CO_2_. Compact spheroids formed within four days. Forty spheroids were collected in a 24-well ultra-low attachment plate (Corning) in HepaRG medium containing 1 nM insulin. The plate was placed on a 3D rotator (PS-M3D; Grant, Shepreth, UK) for one day before transfer to the culture compartments of the 2-OC.

### Real-time quantitative PCR

Liver spheroids were collected for RNA isolation using the NucleoSpin RNA Kit (Macherey-Nagel, Berlin, Germany). cDNA was synthesized by reverse transcription of 200 ng total RNA using the TaqMan® Reverse Transcription Kit (Thermo Fisher Scientific, Waltham, USA). Quantitative PCR was performed using the QuantStudio 5 Real-Time PCR System (ThermoFisher Scientific) and the SensiFAST SYBR Lo-ROX Kit (Bioline, Luckenwalde, Germany), according to the manufacturer’s instructions. The real-time qPCR primers were as follows: insulin receptor forward 5′-CGAGAAACTGCATGGTCGCC-3′ and reverse 5′-CACGCCAAAGGACCACATGTC-3′ and TATA-box-binding protein forward 5′-CCTTGTGCTCACCCACCAAC-3′ and reverse 5′-TCGTCTTCCTGAATCCCTTTAGAATAG-3′.

### Insulin Sensitivity

HepaRG cells were seeded confluently (200,000 cells/cm^2^) in 12-well plates and maintained, as described previously, for eight days. Stellate cells were seeded at a density of 13,000 cells/cm^2^ in 12-well plates and maintained for four days. Liver spheroids were formed as described above. One day before performing the assay, liver spheroids and monolayer cultures were washed three times with phosphate buffered saline (PBS; Corning) and cultured in assay medium, consisting of Williams E (PAN-Biotech) with 5.5 mM glucose and 0.1% bovine serum albumin (Serva, Heidelberg, Germany), overnight. The following day, cultures were incubated in assay medium with 1 nM or without insulin (PAN-Biotech) for 30 minutes at 37 °C. Reactions were stopped by washing cultures three times with 1.5 ml ice-cold PBS on ice. Subsequently, the cells were lysed in 100 µl cell extraction buffer (FNN0011; Thermo Fisher Scientific) containing 1 mM PMSF protease inhibitor (Thermo Fisher Scientific) and protease inhibitor cocktail (P8340; Sigma-Aldrich) for 30 min (monolayer) or 60 min (spheroids). Cell extracts were snap frozen and centrifuged at 13,000 g for 10 min at 4 °C after thawing. Protein concentrations in supernatants were determined using the Pierce BCA protein assay kit (Thermo Fisher Scientific). The ratios between phosphorylated and unphosphorylated AKT were quantified using a human AKT ELISA kit (KHO0111; Therma Fisher Scientific) and a human phospho-AKT pS473 ELISA kit (KHO0111; Thermo Fisher Scientific), as recommended by the manufacturer.

### Co-culture of pancreatic islet microtissues and liver spheroids in the 2-OC

The 2-OC cultures were conducted in standard HepaRG culture medium without insulin, in short including William’s Medium E (11 mM glucose), 10% foetal calf serum, 2 mM L-glutamine, 5 × 10^−5^ M hydrocortisone hemisuccinate, 50 µg/ml Gentamycin Sulfate and 0.25 µg/ml Amphotericin B. Prior to transfer, the organ equivalents were washed twice with PBS/0.1% BSA and equilibrated for 2 h in co-culture medium. Forty liver spheroids and ten pancreatic islet microtissues were loaded into the spatially separated culture compartments of a common circuit. Additional circuits were loaded with either 40 liver spheroids solely or 10 pancreatic islet microtissues solely, for comparison. Each culture compartment was filled with 300 µl medium. A complete medium exchange in both compartments was performed after 24 h of culture. Subsequently, the medium was renewed completely every 48 h. Cultures were ended after 15 days of culture and organ equivalents were collected from the culture compartments.

### *In vitro* glucose tolerance test

To perform an *in vitro* glucose tolerance test, medium from both culture compartments was completely removed and replaced with 315 µl medium, respectively, containing 11 mM glucose. Samples of 15 µl were collected immediately from both liver and islet compartments and pooled to obtain sufficient sample volume for the subsequent analyses. The procedure was repeated for sample analyses following 8, 24 and 48 h dynamic cultivation. This all leads to a maximum volume reduction of 10%, as a complete medium exchange was carried out directly after taking the sample at 48 h. The total area under the glucose and insulin curves was calculated using the trapezoidal method.

### Glucose-stimulated insulin secretion

Eleven islet microtissues per condition (n = 2–4 islet microtissues per circuit) were removed from the culture compartment and collected in separate wells of a GravityTRAP^TM^ plate (InSphero) to analyse the islet functionality by GSIS. After an initial equilibration for 2 h in low glucose buffer (2.8 mM), the islet microtissues were washed and sequentially incubated in 50 µl low glucose buffer (2.8 mM) and then high glucose buffer (16.8 mM) for 2 h, respectively. The buffers were collected after incubation and analysed using a human Insulin ELISA kit (10-1113-01; Mercodia, Uppsala, Sweden).

### Analyses of medium samples

Medium samples were collected during a complete medium exchange every 48 h for glucose, insulin and albumin analyses. Additionally, samples were taken after 0, 8, 24 and 48 h on day 1, 7 and 13 to measure the response to a high glucose load.

Glucose concentrations were measured using the GLU 142 kit (Diaglobal, Berlin, Germany), according to the manufacturer’s instructions, with minor modifications: 95 µl of the reagent was mixed with 5 µl of prediluted sample and incubated for 20 min at room temperature. Absorbance was measured at 520 nm using a standard curve prepared with Williams E containing 11 mM glucose as a reference.

Commercially available ELISA kits were used to measure the insulin (Mercodia) and albumin (E80-129; Bethyl Laboratories, Montgomery, Tx, USA) concentrations. Absorption was determined using a microplate reader at 450 nm. Albumin production was calculated by multiplying the albumin concentration with the remaining volume in the compartment after 48 h of culture.

### Immunohistochemistry

Liver spheroids and pancreatic islet microtissues were embedded in Tissue-Tek® O.C.T. compound (Sakura Finetek, Torrance, CA, USA) for immunohistochemistry. Central cryostat sections of 12 µm were fixed in acetone at −20 °C for 10 min, washed with PBS and blocked with 10% (v/v) goat serum in PBS for 20 min. The pancreatic islet microtissues were immunostained with guinea pig anti-insulin antibody (Dako, Glostrup, Denmark) and mouse anti-glucagon (Abcam, Cambridge, UK) overnight, washed with PBS and, subsequently, developed by goat anti-guinea pig CF594 (Biotium, Fremont, CA, USA) and goat anti-mouse Alexa-488 (Life Technologies, Carlsbad, Ca, USA) for 45 min. DAPI was added for nuclei staining. The same procedure was carried out for the liver spheroids using mouse anti-cytokeratin 8/18 (Santa Cruz, Heidelberg, Germany), rabbit anti-vimentin (Santa Cruz), goat anti-albumin FITC (Bethyl Laboratories, Montgomery, Tx, USA) and mouse anti-Cyp3A4 (Santa Cruz) as primary antibodies and goat anti-mouse Alexa-488 (Life Technologies, Carlsbad, Ca, USA), goat anti-rabbit CF594 (Biotium) or goat anti-mouse CF594 (Biotium) as secondary antibodies. Images were obtained using a Keyence fluorescence microscope

### Statistical analysis

Differences among groups were analysed using unpaired two-tailed Student’s t test or ANOVA as appropriate, and a p value < 0.05 was considered statistically significant.

### Data availability statement

The datasets generated during and/or analysed during the current study are available from the corresponding author on reasonable request.
