# Publisher Correction: Functional coupling of human pancreatic islets and liver spheroids on-a-chip: Towards a novel human ex vivo type 2 diabetes model

**DOI:** 10.1038/s41598-018-20340-1

**Published:** 2018-01-23

**Authors:** Sophie Bauer, Charlotte Wennberg Huldt, Kajsa P. Kanebratt, Isabell Durieux, Daniela Gunne, Shalini Andersson, Lorna Ewart, William G. Haynes, Ilka Maschmeyer, Annika Winter, Carina Ämmälä, Uwe Marx, Tommy B. Andersson

**Affiliations:** 1 TissUse GmbH, Berlin, Germany; 2 Bioscience Diabetes, Cardiovascular and Metabolic Diseases, IMED Biotech Unit, AstraZeneca, Gothenburg Sweden; 3 DMPK, Cardiovascular and Metabolic Diseases, IMED Biotech Unit, AstraZeneca, Gothenburg Sweden; 4 Drug Safety and Metabolism, IMED Biotech Unit, AstraZeneca, Cambridge UK; 5 Department of Physiology and Pharmacology, Section of Pharmacogenetics, Karolinska Institutet, Stockholm, Sweden

Correction to: *Scientific Reports* 10.1038/s41598-017-14815-w, published online 03 November 2017

The original HTML version of this Article contained a typographical error in the publication date ‘03 November 2017’ which was incorrectly given as ‘06 November 2017’. This has now been corrected in the HTML version of the Article.

